# Ileocolic Intussusception in a Leukemic Adult Patient: A Case Report and Review of the Literature

**DOI:** 10.1155/2016/3972605

**Published:** 2016-10-20

**Authors:** Ayoub Innabi, Wa'el Tuqan, Alia Alawneh, Alaa Saleh, Kamal Alrabi, Lina Marei

**Affiliations:** ^1^University of Arkansas for Medical Sciences, Little Rock, AR, USA; ^2^University of New Mexico, Albuquerque, NM, USA; ^3^Tawam Hospital, Al Ain, UAE; ^4^King Hussein Cancer Center, Amman, Jordan

## Abstract

We present a rare case of intussusception in a 41-year-old man with acute myeloid leukemia without an evidence of leukemic infiltration of the bowel. The patient presented to the emergency room with right lower quadrant pain. Initially he was diagnosed with typhlitis. CT scan was done and showed ileocolic intussusception without a definitive lead point identified. Patient underwent hemicolectomy and histopathological study of the specimen did not show any leukemic infiltrate. High suspicion of intussusception should be kept in mind with leukemic patients presenting with abdominal pain.

## 1. Introduction

The gastrointestinal system can undergo various pathological changes in leukemia produced by the disease process or by its treatment. Leukemic gastrointestinal sequela can include leukemic infiltration, infections, hemorrhagic necrosis, and related surgical complications [1]. Various causes of right lower quadrant pain in leukemic patients are reported in literature, and early diagnosis and treatment are essential for survival [2, 3].

We report a case of intussusception in an adult patient with acute myeloid leukemia presenting with right lower quadrant pain. Although this is an uncommon complication in adults, it should be suspected in patients with acute leukemia presenting with abdominal pain.

## 2. Case Presentation

A 41-year-old male patient presented to our hospital in January 2010, with left tonsillar mass, and a biopsy revealed a malignant lymphoma (DLBCL). There were no metastatic lesions except for few left cervical lymph nodes. The patient had a stage IAE DLBCL. He received chemotherapy which was followed by radiotherapy to the left tonsillar region and a complete response was achieved. In 2012, the patient was evaluated for easy fatigability. He was found to have therapy-related acute myeloid leukemia/myelodysplastic syndrome (AML/MDS) and was treated with multiple lines of chemotherapy and he achieved complete remission in January 2014. Six months later he was found to have AML/MDS in relapse. Patient was started on palliative azacitidine.

In September 2014, he presented to the emergency room complaining of right lower quadrant abdominal pain, sudden in onset, moderate in severity, nonradiating, and associated with fever, chills, vomiting, and nonbloody diarrhea. Vital signs were significant for a heart rate of 122 beats per minute and a temperature of 38.3°C (102.4°F). On examination, he looked pale, fatigued, and distressed. He had conjunctival pallor, lungs were clear to auscultation, and his abdomen was soft and nondistended, with moderate right lower quadrant tenderness and no signs of peritoneal irritation. There were no palpable masses.

Laboratory studies showed a white blood count of 1,600 cells/*µ*L, with absolute neutrophilic count of 320 neutrophils/*µ*L. Hemoglobin level was 7.0 g/dL, and a platelet count was 1,000 platelets/*µ*L. Chemistry and electrolytes panel showed no abnormality. Stool analysis was only positive for few red blood cells.

**Figure 1:**
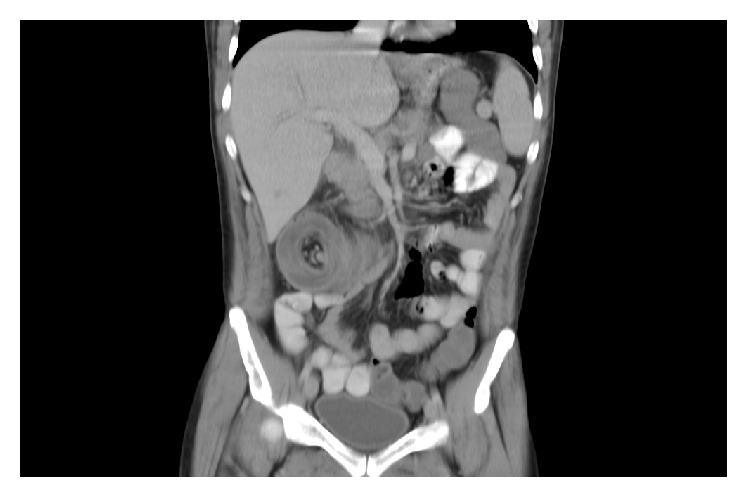
Coronal reconstruction of contrast enhanced abdomen and pelvis CT showing long segment of ileocolic intussusception with classic target/“doughnut” sign.

Initially the patient was diagnosed with febrile neutropenia with suspected typhlitis. Blood cultures were drawn and he was started on broad spectrum IV antibiotics. CT scan of the abdomen and pelvis was ordered and showed ileocolic intussusceptions (Figure 1) with no definitive lead point identified and no signs of upstream bowel obstruction. Over the next few hours, the abdominal pain worsened, and the patient developed bloody diarrhea. The patient underwent right hemicolectomy with end ileostomy. Histopathology results of the removed specimen confirmed an intussuscepted segment of small bowel, which demonstrated a spectrum of changes from mucosal ischemia/infarction to transmural hemorrhagic infarction. There was no evidence of malignancy or any other pathological nidus. The patient hospital stay was complicated by delayed wound healing. Eventually the patient general condition worsened and he passed away after 36 days of the operation.

## 3. Discussion

Intussusception in leukemia patients has almost exclusively been reported in the pediatric population but rarely reported in adult patients [4, 5]. We have found three case reports each describing a case of an adult leukemic patient who developed intussusception [4, 6, 7]. The previous case reports described clinical presentation of intussusceptions in adults with acute myeloid leukemia in which leukemic infiltration of the bowel acted as a lead point for the intussusceptions. In our case, we could not find any evidence of leukemic infiltration on imaging or pathological studies.

Compared to the pediatric population, intussusception is uncommon in adult population, contributing only to 5% of all intussusceptions [8]. Most of the cases of intussusceptions in infants are idiopathic, but in adults around 90% of the cases have a causative factor in which malignancy contributes to nearly 65% of cases [9]. In case of adult intussusception where a lead point cannot be found, intussusception is thought to be idiopathic or secondary to a disease process contributing to dysrhythmic peristalsis of the gastrointestinal tract [10]. Any lesion or irritant in the bowel wall that alters the peristaltic movement may initiate invagination and lead to intussusception [11]. Inflammation of the bowel as a result of neutropenia may have precipitated invagination in our case.

Another important differential diagnosis for acute right lower quadrant abdominal pain in patients with leukemia is necrotizing enterocolitis (NEC). Necrotizing enterocolitis (typhlitis) is a necrotizing process that usually affects the ascending colon, cecum, and terminal ileum. Majority of cases involve hematological conditions, and 2.6% of patients with acute leukemia develop typhlitis [12, 13]. Patients with NEC present with fever in most cases, right lower quadrant pain, diarrhea, nausea, vomiting, and signs of sepsis [12, 14]. Besides being neutropenic, our patient presented to the emergency room with right lower quadrant pain, fever, and vomiting which raised a high suspicion for NEC.

Because of the variety of pathological processes that may cause right lower quadrant abdominal pain in immunocompromised patients, reaching an accurate diagnosis may be problematic. Merine et al. reported usefulness of using CT scan in differentiating the various causes of right lower quadrant abdominal pain in the clinical setting in the immunocompromised patients [2]. It can help to define the location, the nature of the mass, and its relation to the surrounding tissues [15]. In a previous study, abdominal CT has been able to distinguish between intussusception lacking a lead point (no signs of proximal bowel obstruction, target-like or sausage-shaped mass, or layering effect) and that with a lead point (signs of bowel obstruction, bowel wall edema with loss of the classic three-layer appearance) [16]. CT of the abdomen is currently considered the best diagnostic modality to diagnose intussusceptions [17].

Treatment of adult intussusception almost always requires surgical intervention [18]. Due to the high association of adult intussusceptions with malignancy, surgical resection is recommended rather than preoperative pneumatic or hydrostatic reduction [9]. In our case, it was thought there was leukemic infiltration of the bowel; however pathological report showed no leukemic infiltration.

## 4. Conclusion

We described an interesting case of ileocolic intussusception in a leukemic patient without an evidence of leukemic infiltration of the bowel. Although it is a rare cause in adults, it should be kept on the differential of a patient presenting with abdominal pain. CT scan is a successful imaging study that can help in reaching a correct diagnosis.
